# The Interactions Between Diet and Gut Microbiota in Preventing Gestational Diabetes Mellitus: A Narrative Review

**DOI:** 10.3390/nu16234131

**Published:** 2024-11-29

**Authors:** Luiza-Andreea Beldie, Cristina-Camelia Dica, Maria Moța, Bianca-Florentina Pirvu, Marilena-Alexandra Burticală, Adina Mitrea, Diana Clenciu, Ion Cristian Efrem, Beatrice Elena Vladu, Diana Cristina Protasiewicz Timofticiuc, Maria Magdalena Roșu, Theodora Claudia Gheonea, Anca Maria Amzolini, Eugen Moța, Ionela Mihaela Vladu

**Affiliations:** 1 Department of Diabetes, Nutrition and Metabolic Diseases, County Clinical Emergency Hospital of Craiova, 200642 Craiova, Romania; drluizabeldie@yahoo.com (L.-A.B.); drcristinadica@yahoo.com (C.-C.D.); pirvubianca178@gmail.com (B.-F.P.); burticalaalexandra@gmail.com (M.-A.B.); diana_protasiewicz@yahoo.com (D.C.P.T.); 2 Doctoral School, University of Medicine and Pharmacy of Craiova, 200349 Craiova, Romania; mmota53@yahoo.com (M.M.); eugenmota@yahoo.com (E.M.); 3 Department of Diabetes, Nutrition and Metabolic Diseases, Faculty of Medicine, University of Medicine and Pharmacy of Craiova, 200349 Craiova, Romania; diana.clenciu@umfcv.ro (D.C.); theodora.gheonea@umfcv.ro (T.C.G.); ionela.vladu@umfcv.ro (I.M.V.); 4 Department of Medical Semiology, Faculty of Dentistry, University of Medicine and Pharmacy of Craiova, 200349 Craiova, Romania; 5 Faculty of Medicine, University of Medicine and Pharmacy of Craiova, 200349 Craiova, Romania; beatricevladu75@gmail.com; 6 Department of Diabetes, Nutrition and Metabolic Diseases, Faculty of Midwives and Nursing, University of Medicine and Pharmacy of Craiova, 200349 Craiova, Romania; maria.rosu@umfcv.ro; 7 Department of Medical Semiology, Faculty of Medicine, University of Medicine and Pharmacy of Craiova, 200349 Craiova, Romania; anca.amzolini@umfcv.ro

**Keywords:** gestational diabetes mellitus, gut microbiota, probiotics, diet

## Abstract

Recent studies have revealed that dysbiosis, defined as alterations in gut microbiota, plays an important role in the development and the progression of many non-communicable diseases, including metabolic disorders, such as type 2 diabetes mellitus and gestational diabetes mellitus (GDM). The high frequency of GDM makes this disorder an important public health issue, which needs to be addressed in order to reduce both the maternal and fetal complications that are frequently associated with this disease. The studies regarding the connections between gut dysbiosis and GDM are still in their early days, with new research continuously emerging. This narrative review seeks to outline the mechanisms through which a healthy diet that protects the gut microbiota is able to prevent the occurrence of GDM, thus providing medical nutritional therapeutic perspectives for the management of GDM.

## 1. Introduction

Gestational diabetes mellitus (GDM) is defined as glucose intolerance, which first appears during pregnancy and is characterized by increased blood glucose levels. Being one of the most common metabolic complications encountered during pregnancy, it has negative effects on the development of the fetus, but also on the mother, leading to negative outcomes both in the short term and in the long term [1,2,3]. According to the data reported in 2019 by the International Diabetes Federation, gestational diabetes affects one in six newborns worldwide [4]. Globally, the prevalence of GDM varies in various population groups, based on different applied diagnostic and screening criteria [5]. It was observed, following a systemic analysis carried out in 2012, that GDM varies between 3.6% and 38% with the Carpenter and Coustan criteria, 1.4% and 50% for the NDDG criteria, 2% and 24.5% using WHO criteria and 2% and 19% based on IADPSG criteria [6].

To support the growth of the fetus, significant metabolic, immune system and hormonal alterations arise during pregnancy [7]. Beginning with the first trimester, the maternal insulin secretion rises, transitioning into an anabolic state which is characterized by fetal development through adipose reserves, inducing gestational weight gain. Metabolic hormones and pro-inflammatory cytokines elevate with the progression of pregnancy and the onset of insulin resistance occurs. As pregnancy proceeds, the maternal blood glucose and free fatty acid (FFAs) arise through catabolic metabolism defined by gluconeogenesis and lipolysis. The placenta is the key organ, with the help of which these nutrients pass into the fetal circulation. Insulin resistance causes pancreatic beta-cell dysfunction in susceptible women, contributing to hyperglycemia [8].

The intestinal microbiome is defined as the multitude of microorganisms that populate the gastrointestinal tract, having the role of a virtual endocrine-metabolic organ involved in the control of the various mechanisms that underlie the harmonious functioning of the body [9,10]. As a result of research, a directly proportional relationship between the intestinal microbiota and the occurrence of GDM was found; therefore, various bacterial species are correlated with the dysfunction of carbohydrate metabolism. In this manner, the intestinal microbiome can be used as a screening method in the early detection of GDM and in the interventions aimed at reducing the risk of GDM [11].

The imbalance of various bacterial populations, together with the reduced microbial variety, is known as intestinal dysbiosis, which is correlated with insulin resistance and the occurrence of GDM [11]. Recent studies have highlighted the fact that reducing the risk of GDM and optimizing glucose metabolism can be achieved through a balanced diet that supports the integrity of intestinal health, rich in dietary fiber, the consumption of probiotics and anti-inflammatory nutrients [12].

**Figure 1:**
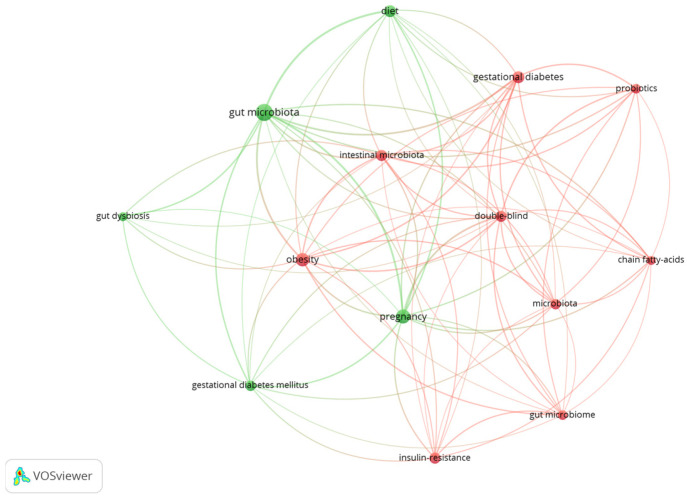
VosViewer (version 1.6.19) bibliometric study of Web of Science papers studying the associations between gestational diabetes mellitus, gut microbiota and dietary factors.

As the gut microbiota has been studied for a relatively short amount of time, with the first studies being conducted starting in 2001, when the term microbiome was used by Joshua Lederberg [13], data regarding GDM and the gut microbiota are still scarce. A literature search was performed on recognized databases (PubMed and Web of Science) using terms such as “gestational diabetes”, “gut microbiota”, “gut dysbiosis” and “diet”. The search was performed by three review authors and the most relevant articles were identified by the most experienced two authors. As Figure 1 illustrates, there is a knowledge gap in the scientific medical literature that will probably be the object of many future studies.

Taking into consideration the facts presented above regarding the limited data on the relationships between GDM and the gut microbiota, we aimed to analyze the mechanisms through which the relationship between the intestinal microbiome and diet prevents the occurrence of GDM in a narrative review. Therefore, this paper seeks to provide a complete perspective on the optimization of carbohydrate metabolism during pregnancy by researching the underlying mechanisms contributing to the modification of the intestinal microbiota through nutritional interventions.

## 2. Pathophysiology of GDM and the Role of Gut Microbiota

The most remarkable change in the maternal metabolism is the onset of insulin resistance. It is essential that the fetus receives nutrients; however, an impaired insulin response might increase the risk of GDM. Various hormonal shifts take place throughout pregnancy leading to elevated levels of humas placental lactogen (hPL), cortisol, growth hormone and progesterone, which counteract insulin function. These are counter-regulation hormones that assure adequate blood sugar levels, in the maternal circulatory system, for fetal nourishment [14,15]. During the first phase of pregnancy, the anabolic state increases the insulin sensitivity ensuring efficient glucose uptake by adipose tissue. This helps accumulate energy reserves for the later stages of pregnancy [15]. The weight gain in the mother is determined by amplified insulin function and this assures energy is preserved for the fetus as gestation continues [16].

The onset of the catabolic state in the second and third trimester contributes to the transition from reduced insulin sensitivity to increased insulin resistance; therefore, less glucose is used by the maternal tissue and more is used for the growing fetus [17]. Studies have indicated that insulin sensitivity can diminish by up to 50–60% in the late phase of pregnancy [16]. Increasing amounts of hormones such as hPL and progesterone are produced by the placenta as the pregnancy progresses, reducing the absorption of glucose by the maternal cells. Through this mechanism, the fetus benefits from the glucose circulating in the maternal blood [16,18]. Towards the end of pregnancy, lipolysis intensifies [19] enhancing the level of FFAs in the bloodstream, promoting the appearance of beta cell dysfunction and further decreasing insulin sensitivity [16].

Insulin resistance is amplified by the persistent low-intensity inflammation that occurs during pregnancy. This leads to the increase in pro-inflammatory cytokines TNF-alpha, IL-6 and *C*-reactive protein that interfere with the action of insulin by reducing the function of insulin receptor substrates [20,21]. The requirement for nutrients for the development of the fetus increases gradually as the pregnancy progresses, and in this manner the placenta becomes an endocrine organ which secretes hormones that enhance insulin resistance [16]. During pregnancy, the pancreas produces more insulin to compensate for marked insulin resistance, thus maintaining a state of maternal euglycemia [15]. On the contrary, in GDM, hyperglycemia is the result of the inability of beta cells to fulfill the increased insulin requirements [12].

Systemic inflammation, energy balance and insulin sensitivity are influenced by the composition of the intestinal microbiome, which is essential for maintaining metabolic health [22]. Short chain fatty acids (SCFAs), such as acetate, propionate and butyrate, are an essential element in metabolic regulation. They are produced by the fermentation of dietary fibers at the intestinal level and are considered to reduce inflammation and improve glucose metabolism by regulating the secretion of intestinal hormones and inhibiting proinflammatory cytokines [23,24]. Inflammation is also prevented by maintaining the integrity of the intestinal barrier, and thus harmful bacteria and their metabolites cannot be translocated into the bloodstream [25].

An essential role in metabolic health is played by bile acids that facilitate the digestion and absorption of fats. By activating nuclear receptors, including the Farnesoid X receptor (FXR), they influence energy metabolism and insulin action [26]. Bile acids are modified by the intestinal microbiome, these changes can contribute to insulin resistance and obesity [27]. A greater microbial variety is correlated with better insulin sensitivity, a lower level of systemic inflammation and better metabolic outcomes, decreasing the risk of metabolic syndrome [28,29]. The diversity of the intestinal microbiome is considerably modified during pregnancy, being characterized by increased Proteobacteria and Actinobacteria species [30].

Changes in metabolism during pregnancy are associated with modifications in the composition of the intestinal microbiome, indicating that pregnant women with a greater abundance of Lactobacillus and Bifidobacterium have an enhanced insulin sensitivity in contrast to those with a less varied microbiota [31]. Studies have shown that a reduced diversity of intestinal bacteria with an increase in pro-inflammatory ones can lead to GDM. An imbalance in the intestinal microbiome leads to insulin resistance and systemic inflammation, which are main elements in the pathogenesis of GDM [11]. A systemic review published in 2021 by Kunasegaran et al. emphasizes the importance of intestinal microbiota balance; the alteration of this equilibrium was correlated with weight gain and hyperglycemia [32]. A diet rich in fiber has been shown to prevent the occurrence of dysbiosis and metabolic disorders related to pregnancy, highlighting the correlation between changes in the microbiome and the state of the health of the mother during pregnancy [33].

By altered functional pathways that contribute to hyperglycemia and insulin resistance, favoring weight gain through metabolic processes and microbial alterations that worsen inflammatory imbalances, the gut microbiota plays an important role in the pathophysiology of GDM.

**Figure 2:**
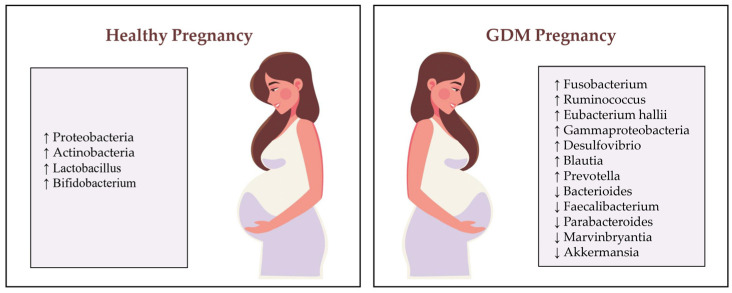
The gut microbiome profile in healthy pregnancy versus gestational diabetes mellitus [30,31,34,35,36,37,38]. ↓ Decrease in the number of species, ↑ Increase in the number of species.

Diverse microbiome groups modifications are observed in patients with GDM, for instance, enhanced levels of *Fusobacterium* [34], *Ruminococcus* [35], *Eubacterium hallii* [36], *Gammaproteobacteria* [37], *Desulfovibrio* [35], *Blautia* [36] and *Prevotella* [38], and decreased concentrations of Bacterioides [38], *Faecalibacterium* [34], *Parabacteroides* [38], *Marvinbryantia* [35] and *Akkermansia* [38] (Figure 2).

The role of different bacteria has been studied, demonstrating their relationships with the metabolic profile and inflammatory response. Prevotella can enhance intestinal barrier dysfunction [39] by promoting the connection between branched-chain amino acids and impaired insulin sensitivity [40]. The outer membrane of *Gammaproteobacteria* consists in lipopolysaccharides that release an endotoxin responsible for contributing to a gastrointestinal inflammatory state [41].

In 2019, the research published by Ye et al. explored the connections between blood glucose metabolism and gut microbiota. Findings indicated that the increased preponderance of the *Blautia* and *Eubacterium hallii* group revealed a proportional relationship with blood glucose levels [36]. In contrast, *Faecalibacterium* was associated with a decrease in glucose levels by undergoing fermentation to generate SCFA, particularly butyrate [42]. A deficiency in *Faecalibacterium*, as well as a simultaneous lack of butyrate impairs the mitochondrial function of colonocytes leading to autophagy and increased intestinal permeability. Furthermore, an inadequate level of butyrate decreases the proliferation of pancreatic beta cells, causing disrupted glucose metabolism [43,44,45].

Reuterin, synthesized during the fermentation of glycerol by the *Eubacterium hallii* group, is a potent antimicrobial metabolite that fulfils a significant role in modulating oxidative stress involved in the pathogenesis of GDM [46,47,48]; thus, the important role that the intestinal microbiome has in the development of GDM can be highlighted.

## 3. Dietary Modulation of Gut Microbiota and Its Impact on Glucose Metabolism

### 3.1. Dietary Patterns and Gut Microbiota

The gut microbiota is a remarkable environment. It interacts with a person’s diet, influencing a wide range of metabolic processes and, consequently, a number of conditions, one of which is the body’s response to glucose, which can determine GDM [49]. A blend of genetic, social, environmental, cultural, economic, health and lifestyle factors shape people’s eating behaviors, and therefore, recent studies on the microbiota were centered on dietary patterns rather than a traditional analysis of macro- and micronutrients, bringing up the idea that people eat complex diets, not individual nutrients [50,51]. The distinction between dietary patterns and short-term dietary habits is fundamental in establishing the risk of chronic disease, in accordance with long-term exposure to a diet pattern, rather than centering attention on individual nutrients [52].

The human gut is home to over 250 different species of bacteria, fungi, viruses and archaea, representing a complex system that evolves throughout a person’s life and that serves as home for around 10^13^ bacterial cells [53,54]. At birth, the gut microbiota community begins to establish itself and is influenced by various factors, including the type of delivery, feeding method, antibiotic use and genetic inheritance as well as several prenatal factors, such as the mother’s dietary patterns, obesity status and smoking habits [55,56]. In the early years, this microbiota undergoes significant diversification and is crucial for the development of the immune system, and also plays a key role both in children and in adults in regulating intestinal and hormonal functions, detoxification and enhancing the function of the intestinal barrier [57,58,59,60,61].

Human microbiota consists mostly of five phyla of bacteria, including the Firmicutes (60% to 80%, composed of the classes *Clostridia*, *Bacilli* and *Negativicutes*, including Gram-negative genres), the Bacteroidetes (20% to 40% including the classes *Bacteroidia*, *Flavobacteria*, *Sphingobacteria* and *Cytophagia*, with only Gram-negative genres), the Proteobacteria, the Actinobacteria and the Verrucomicrobia, and one Archaea phyla, the Euryarchaeota [62]. Typically, restricted anaerobes (such as *Bacterioides*, *Clostridium*, *Eubacterium*, *Ruminococcus*, *Peptococcus*, *Fusobacterium* and *Bifidobacterium*) prevail over facultative anaerobic genera (such as *Lactobacillus*, *Escherichia*, *Enterobacter*, *Enterococcus*, *Proteus* and *Klebsiella*), with *Cyanobacteria*, *Fusobacteria* and *Spirochaeataceae* being less predominant [62]. Eubiosis describes a balanced gut microbial ecosystem with a predominance of beneficial bacteria; therefore, dysbiosis disrupts and interferes with the normal activity of the gut, being linked to the onset of various diseases [63].

Different dietary patterns impact differently the composition of the gut microbiota, and have a substantial impact on human health. A Western diet, high in animal fats and refined sugars, low in fiber and with frequent snacking, leads to a prolonged postprandial state which is associated with dysbiosis, the development of low-grade systemic inflammation and, consequently, higher risks of diabetes mellitus, insulin resistance, dyslipidemia, inflammatory bowel disease, neoplasms and cardiovascular diseases, including atherosclerosis, cardiomyopathy, hypertension and heart failure [62,64]. One of the core characteristics of Western diet is the low consumption of nutrient-dense foods, namely fruits, vegetables and whole-grains, that limits the growth of beneficial bacteria, like *Bifidobacteria* and *Lactobacillus*, leading to a reduction in microbial diversity [65]. Furthermore, high-fat, high-sugar and highly processed foods create a proper environment for pro-inflammatory bacteria, including strains from the Proteobacteria phylum, to develop and disrupt gut bacterial function [66]. Decreased fiber consumption leads to decreased short chain fatty acids production, and short chain fatty acids are crucial for metabolic health; their deficit causes systemic inflammation, reduced insulin sensitivity and alterations in glucose metabolism [67]. Dysbiosis can damage the intestinal epithelium, allowing lipopolysaccharides from certain bacteria to enter the bloodstream and cause endotoxemia [68]. All of these changes contribute to a state of systemic inflammation that triggers multiple hormonal imbalances and promotes insulin resistance, exacerbating the risk of GDM. [69]

In contrast, a Mediterranean diet, rich in fruits, vegetables, whole grains and olive oil, is associated with greater microbial diversity, having a considerable amount of beneficial bacteria such as *Bifidobacterium* and *Bacterioides* species and a reduced growth of *Firmicutes* and *Blautia* species, and thus, is linked to improvements in inflammatory and oxidative conditions, and better metabolic health [70]. Other dietary patterns that are also highly beneficial for the human microbiota are plant-based diets (vegetarian and vegan), defined by their high-fiber and low-fat content, which allows bacteria like *Prevotella* and *Bacteroides* to grow, while inhibiting the development of *Firmicutes* species [71]. The fiber-rich components of these dietary patterns foster a stable gut microbiome, with an enriched and diverse microbiota [70]. This robust microbial community ferments fiber into SCFAs, including butyrate, acetate and propionate, that mitigate inflammation, fortify gut barrier integrity and modulate metabolic processes, further contributing to better insulin sensitivity and enhanced glucose control [72]. What is more, olive oil, a cornerstone of the Mediterranean diet, is rich in polyphenols and antioxidants that may suppress inflammatory bacteria and generate bioactive metabolites that endorse immune function and modulate inflammation [73]. These dietary patterns enhanced glucose regulation, reduced systemic inflammation and balanced the microbial profile and weight control, offering unique advantages for preventing and managing gestational diabetes mellitus and having multifaceted benefits for maternal and fetal health.

### 3.2. Mechanisms of Dietary Modulation

The potential of nutritional adjustments in the prevention of a wide spectrum of diseases has an unlimited, but at the same time, unfulfilled potential, as a consequence of limitations in understanding the complex diet–microbiome interactions [51]. Carbohydrates are divided into digestible carbohydrates and fibers. Monosaccharides, disaccharides and polysaccharides are digestible carbohydrates that, with the help of the body’s enzymes are broken down in the small intestine, producing glucose that is absorbed into the bloodstream and used for energy [74]. When consumed in high quantities, they can reach the colon, where they are rapidly utilized by fast-fermenting microbes, for example *Enterobacteriaceae* spp. that may proliferate disproportionally and incline the microbiome balance in favor of less beneficial species [75].

Fibers (lignin, cellulose, hemicellulose, glucans, gums, resistant starches, pectins, etc.) are non-digestible oligosaccharides which remain intact as they pass through the digestive system but once they reach the human gut, saccharolytic microbial fermentation begins, producing SCFAs and gases [74,76]. Microbiota composition as well as dietary fiber intake influences the types and amounts of SCFAs generated, because each microbial species has unique characteristics and releases distinct metabolites, their growth being influenced by the dietary content of non-digestible complex carbohydrates [74,77]. High fiber diets generally promote the growth of beneficial bacteria such as *Bifidobacterium* and *Lactobacillus* which further contribute to gut health by maintaining a balanced gut pH and reducing inflammation. SCFAs also act as biochemical messengers; they bind to the intestinal L-cells and ignite the release of glucagon like peptide 1 and peptide YY and support the gut–brain axis [78]. Specific bacteria, such as *Bifidobacterium*, *Lactobacillus*, *Faecalibacterium*, *Eubacterium* and *Roseburia* can produce significant amounts of SCFAs, which can provide a wide range of health benefits such as lowering systemic inflammation, strengthening the intestinal barrier and helping regulating glucose levels and appetite, hence, being essential for the gut microbiota [79].

Proteins are broken down into peptides and amino acids through a multi-step process that begins in the stomach and continues in the small intestine. The small intestine hosts a unique microbiota, with lower bacterial density compared to the colon, which includes facultative anaerobes such as *Lactobacillus* and *Streptococcus* that release metabolites form the amino acids [80]. In the colon, undigested proteins and amino acids suffer biochemical processes, proteolysis, deamination and transamination, induced by bacteria from the genera *Clostridium*, *Bacteroides*, *Enterobacterium*, *Bifidobacterium*, and *Lactobacillus*, and are transformed into metabolites, including SCFAs and ammonia, and produce phenolic compounds such as *p*-cresol, phenylpropionate (from tyrosine), phenylacetate (from phenylalanine), indole propionate and indole acetate (from tryptophan) [81]. Diets high in protein enhance resident microbiota metabolic activity and create an oxygen-poor environment in the gut that supports the activity of anaerobic gut species like *Clostridia* and *Fusobacteria* to metabolize amino acids [82].

Ammonia may act as a tumor promoter in the gut, and amines, which result from the decarboxylation of amino acids and peptides, may serve as precursors for the formation of nitrosamines, which are recognized carcinogens found in human feces [83]. Some amino acids like glycine, threonine, lysine, aspartate, alanine and glutamate can be fermented by the gut microbiota and produce SCFAs which, as stated above, play an important role in supporting the intestinal barrier, regulating inflammation pathways and modulating the gut microbiota [84]. Moreover, butyrate inhibits specific enzymes involved in epigenetic regulation and can induce apoptosis in various cancer cells [85]. Each protein source, plant or animal, impacts differently the gut microbial community. Animal-based proteins tend to promote the growth of certain bacteria genera like *Bacteroides* and *Clostridium*, which exert beneficial effects in moderate amounts, but when they are present in excess they can produce metabolites like branched-chain fatty acids and ammonia that have been acknowledged to negatively impact gut barrier integrity [86]. Plant proteins can reshape the gut microbiota by encouraging the proliferation of beneficial bacteria, for instance *Bifidobacterium*, that raise the production of SCFAs, thus lowering the gut pH and inhibiting the development of pathogenic species like *Enterobacteria* and *Clostridium* [84]. Having said that, a key to balanced gut microbiota is incorporating both plant- and animal-based proteins, as a varied and moderate protein intake sustains microbiota stability. In contrast, inadequate intake may jeopardize the maternal microbiome, with severe consequences for the development of the future child, both in weight and cognitive development [87].

High-fat diets can lead to dysbiosis, characterized by an increase in Gram-negative bacteria that contain lipopolysaccharides (LPS) in their outer membranes. LPS bind to toll-like receptor 4 (TLR-4), triggering the inflammatory cascade and release of pro-inflammatory cytokines such as TNF-α, IL-1 and IL-17, which disrupts the vagal feedback mechanism that regulates food intake, contributes to local inflammation and increases the intestinal barrier permeability, allowing LPS to enter the bloodstream, thus, developing a chronic inflammation state [79,83,88]. Medium-chain and long-chain fatty acids are saturated fatty acids that tend to reduce microbial diversity and increase inflammatory bacteria like *Firmicutes* and *Bacteroides* and are correlated to the development of metabolic diseases [89]. On the other side of the coin, monounsaturated fatty acids have multiple beneficial effects not only from a metabolic perspective but also on gut microbiota composition, creating a beneficial environment for the growth of *Bifidobacterium* and *Lactobacillus* spp. [89]. On top of that, polyunsaturated fatty acids, especially omega-3 fatty acids, have anti-inflammatory properties, improving the abundance of fruitful microbiota [89].

Not only does the gut microbiota influence the intestinal epithelium, but it also influences the whole body, with an important impact on host cells and their physiology, as it was demonstrated in recent research that linked imbalances in gut microbiota composition with chronic diseases such as metabolic disorders, cancer and inflammatory bowel disease (IBD) [90]. This relationship is further explored through the lens of epigenetic regulation, which includes mechanisms that can ultimately cause heritable changes in a cell’s phenotype without altering the underlying DNA sequence [91]. This dynamic ecosystem exerts a wide array of functions, especially pathways for the metabolism of dietary macromolecules and the capacity to metabolize phytochemicals, through extensive cross-feeding networks and with a different range of possible outcomes depending on the species present, confirming the wealth of metabolic functionality encoded within the gut microbiome [92].

### 3.3. Nutritional Interventions in Pregnancy

Managing nutrition in pregnancy involves a well-balanced diet that meets the nutritional needs both for the mother and the fetus while keeping in mind the dynamic shifts in the composition and diversity of maternal gut microbiota. Nutritional interventions in pregnancy are a key consideration for optimizing maternal and fetal heath as stated in The Developmental Origin of Health and Disease theory. This theory emphasizes that the perinatal environment is critically influential in the development of mammals, considering that adverse experiences, such as a suboptimal intrauterine environment, can predispose individuals to heightened susceptibility to various diseases in later life [93]. The necessity and scope of these interventions is defined through a comprehensive approach that integrates the nutritional status of the individual, specific conditions encountered during pregnancy and risk factors [94]. While certain nutritional recommendations are beneficial regardless of risk status, some groups of women require targeted interventions [95]. Tailored interventions address disparities in nutrient intake and prevent complications related to malnutrition or overnutrition while encompassing the cultural, social and personal preferences of a pregnant woman, emphasizing the need for nutritional intervention in all of these women [95].

An elevated body mass index prior to and during pregnancy has been linked to insulin resistance and gestational diabetes, significantly increasing the risk of serious complications for both the mother and the fetus, such as pre-eclampsia, perinatal morbidity and a greater likelihood of developing metabolic, cardiovascular and mental health disorders over the course of the next generation’s lives [96,97].

As stated above, dietary interventions impact gut microbial composition in various ways. While high saturated fatty acid diets reduce microbial diversity and richness, diets rich in unsaturated fatty acids increase microbiome diversity. Polyunsaturated fatty acids (PUFAs), particularly omega-3 and omega-6 fatty acids, help modulate the hypothalamic inflammation pathway, being vital for maintaining health and preventing diseases, including those related to cardiovascular health and diabetes mellitus [79,98]. Additionally, consistent adherence to the Mediterranean diet has shown improvements in intestinal barrier integrity, inflammation and insulin sensitivity through gut microbiota-dependent mechanisms, promoting the growth of beneficial microbial species, often contrasting the gut enterotypes observed in studies evaluating the effects of a high-fat diet [99]. The Mediterranean diet has been shown to increase the concentrations of *Lactobacillus* spp., *Bifidobacterium*, *Coprococcus*, *Dorea*, *Eubacterium* and *Lachnospiraceae* and increase levels of *Faecalibacterium prausnitzii*, a gut bacterial species with anti-inflammatory effects [100,101]. Caloric restriction in GDM requires a structured plan that should be done by professionals for ensuring glycemic control while meeting the nutritional demands of pregnancy without impairing fetal growth and maternal ketosis [102].

In accordance with the last ADA recommendations, in GDM management, carbohydrate intake is typically between 35 and 50%, with a minimum of 175 g CH a day depending on each individual’s specific needs, for the prevention hyperglycemia while ensuring sufficient energy for maternal and fetal health. [103]. Carbohydrate intake should be spread throughout the day in three meals and 2–3 snacks and should include low-glycemic index carbohydrates, fiber-rich carbohydrates, whole grains, vegetables and fruits over refined carbohydrates for improving glycemic outcomes and lowering the risk of fetal complications [104]. Proteins help modulate blood sugar levels and carbohydrate absorption and contribute to the state of fullness, thus reducing the risk of overeating. A well-designed dietary plan for GDM typically includes a minimum of 71 g of protein, about 20–25% of daily intake, with a thoughtful balance between animal ad protein sources to maximize their nutritional benefits [103]. In regards to fat intake, the type of dietary fats consumed plays a significant role in regulating blood sugar levels and inflammation. Fats should attain about 20–35% of daily caloric intake with an emphasis on MUFA and PUFA fats while minimizing saturated and trans fats in order for pregnant women to optimize their health and fetal development [105]. Moreover, proper intake of micronutrients ensures optimal metabolic functioning and fetal development. In some cases, supplementation may be needed to meet the physiological demands of pregnancy and support maternal and fetal health.

To complement the dietary plan, all of these interventions should be integrated into a personalized strategy that also includes physical activity, routine glucose monitoring and psychological support

In pregnancy, nutritional interventions are a must in order to shape, in a beneficial way, the gut microbiota by increasing the diversity of beneficial bacteria. Given the fact that there is a great global burden, produced by the high-fat, low-fiber diets causing metabolic disorders such as obesity and gestational diabetes mellitus, significant efforts are made in the study of precision nutrition, not only for restoring these unfavorable metabolic states, but also for the primordial prevention of potential metabolic disorders that may arise in the fetus [79].

## 4. Interactions Between Diet, Gut Microbiota and Gestational Diabetes Mellitus Prevention

The connection between the intestinal microbiota and glucose homeostasis is established by the modulation of immunity, but also by the SCFAs resulting from the activity of the intestinal microbiota and bile acids. Butyrate, propionate and acetate stimulate glucose uptake by peripheral tissues and support the secretion of glucagon-like peptide-1 (GLP-1), which stimulates insulin release and homeostatic glucose control [106]. Diet-induced insulin resistance is prevented by butyrate by optimizing the integrity of the intestinal barrier and decreasing inflammation [107]. Another essential role in carbohydrate metabolism is fulfilled by bile acids synthesized in the liver that can be transformed into secondary bile acids by intestinal bacteria, therefore activating nuclear receptors such as Takeda G-protein-coupled receptor 5 (TGR5) and FXR, receptors which optimize insulin sensitivity and regulate lipid metabolism [108].

Low-grade chronic inflammation, which is directly correlated with insulin sensitivity, occurs as a consequence of an imbalance of the microbiota that leads to the alteration of the intestinal barrier with increased permeability, thus facilitating the entry of endotoxins into the bloodstream, especially lipopolysaccharides (LPS) [68]. These mechanisms can be directly modulated by nutrition. The variety of the intestinal microbiome might be enhanced by using prebiotics and probiotics that can be found naturally in fermented products or yogurt [109,110]. Dietary fibers increase SCFAs production and optimize carbohydrate metabolism and decrease the risk of developing GDM [111]. A study by Huating Li et al. demonstrated that a diet enriched with resistant starch improved glucose tolerance and insulin sensitivity through different mechanisms, such as decreasing the chronic inflammatory response by reducing the levels of pro-inflammatory cytokines, including TNF-α and (IL)-1β [112]. Changes were also observed in metabolites derived from the microbiota, especially secondary bile acids, with an increase in glycodesoxycholic acid, but also in deoxycholic acid, 7-ketolithocholic acid and taurodeoxycholic acid, these acids are linked to increased insulin sensitivity. Thus, it can be stated that resistant starch influences the dynamics and restructures the composition of the intestinal microbiota [113,114,115].

Among the dietary patterns that have been noted for a reduced risk of GDM, are the Mediterranean diet, the low glycemic index diet and the plant-based diet, all of which have a positive impact on glucose metabolism [109,110,111,116,117,118].

The Mediterranean diet is based on the abundance of vegetables and fruits, whole grains and healthy fats, especially olive oil. A study published in 2018 by Assaf-Balut et al. highlighted the positive consequences of the Mediterranean diet on pregnant women. They had a lower incidence of GDM compared to those who did not adopt this diet, by improving the variety of the intestinal microbiome and increasing the generation of short-chain fatty acids that optimize insulin sensitivity [116,117]. Monounsaturated fatty acids (MUFA) found in fatty fish, vegetable oils and nuts are an important part of the Mediterranean diet, and studies have shown that incorporating them into the diet reduces the risk of developing GDM [116]. MUFA have been linked to an increase in beneficial intestinal bacterial cultures, such as Firmicutes, Proteobacteria and Bacteroides, which have been associated with an increased secretion of SCFA; hence, it decreases the incidence of metabolic disorders [89]. Furthermore, it was shown that MUFA reduces chronic inflammation by increasing the secretion of IL-10, promoting the polarization of M2 macrophages and suppression of the NLRP3 inflammasome [119,120,121].

Diets with a low glycemic index are based on the consumption of carbohydrates that cause a slow and moderate increase in the level of blood glucose, lowering the demand for insulin. In 2009, a study published by Moses et al. highlighted the fact that the risk of developing GDM is lower in pregnant women who followed a low GI diet compared to those who adopted a higher GI diet [118]. Through the favorable impact these diets have on the intestinal microbiome, they reduce the occurrence of dysbiosis, which is correlated with inflammation and insulin resistance [122].

Plant-based diets stand out for their emphasis on plant foods that are rich in fiber that, through fermentation, leads to the synthesis of SCFAs, which have been shown to increase insulin sensitivity [123]. An analysis indicated that women who adhered to a plant-based diet might have had a lower incidence of GDM, and these results were correlated with improved gut microbiota [124]. Studies have observed a correlation between supplementing the diet with polyphenols, which are naturally found in vegetables, fruits and teas, and decreasing the risk of developing GDM. The mechanisms by which this phenomenon occurs are complex, the consumption of polyphenols has been associated with the reduction of chronic inflammation, but also of oxidative stress [125]. A minor portion of the polyphenols derived from dietary sources is assimilated at the small intestine, where they play an important role in the integrity of the intestinal barrier and the release of cytokines, immunoglobulins and mucous peptides, but also molecular signaling mechanisms; the remaining portion reaches the large intestine where it is metabolized by the intestinal microbiome, amplifying polyphenol bioavailability and potentiating their effects [126,127,128,129].

The studies have increasingly directed attention toward the importance of probiotics, prebiotics and dietary fibers, which by modulating the intestinal microbiome lead to the improvement of metabolic function and the prevention of GDM. Supplements with probiotics restore the balance of the intestinal microbiota and at the same time decrease systemic inflammation by supporting the integrity of the intestinal mucosa [130]. Prebiotics are non-digestible food components that favor the development of beneficial bacterial species in the intestine, such as *Bifidobacterium*, and contribute to increasing the diversity of the microbiota [109]. A 2014 study by Dehghan et al. revealed that diets characterized by a high content of prebiotics lead to better glycemic control and decrease insulin resistance in pregnant women due to enhanced SCFA synthesis [131]. Dietary fibers from whole grains, vegetables, fruits and legumes support the proliferation of beneficial bacteria, reducing insulin resistance, and thus have an important role in metabolic health [109].

## 5. Clinical Evidence on Diet, Gut Microbiota and GDM

In the last decades, an abundance of studies regarding the pathological mechanisms of GDM have emerged [5,8,132,133,134]. Among the research papers that were published, special attention was paid to microbiome studies in GDM, as well as nutritional interventions in GDM. Table 1 summarizes some of the studies that assessed different nutritional interventions in GDM. All of these studies take into consideration the usefulness of probiotics in managing aspects of metabolic health during pregnancy, especially concerning insulin levels and inflammatory markers. Although the mechanisms through which probiotics influence glucose metabolism remain largely unknown, it was suggested that probiotics can induce benefits by restoring correct microflora, as well as normalizing intestinal permeability and controlling pro-inflammatory mediator secretion [135]. However, as there is still conflicting data, further research is needed to clarify the impact of these compounds on glycemic control and overall pregnancy outcomes.

## 6. Future Directions and Personalized Approaches

### 6.1. Potential for Personalized Nutrition

Medical interventions must be tailored according to a person’s genetic, metabolic and lifestyle characteristics, since this enables a more accurate evaluation of risks and the early identification of potential health issues [145]. Individual responses to nutrients support the idea of the unicity of every individual, as people respond differently to nutrients, particularly macronutrients, and this response influences postprandial glycemia, energy expenditure and blood lipid profiles [146].

Personalized medicine can, by integrating genetic profiling, biomarkers and patient-specific data, refine the nutritional guidelines and medication regimens for preventing the development of diabetes; it can also identify individuals who are at great risk due to genetic factors, differences in insulin sensitivity or distinct metabolic reactions to specific dietary components [145,147]. Designing dietary interventions by taking into consideration the individual’s metabolic response to specific macronutrients as well as lifestyle modifications, to improve insulin sensitivity, represents a cornerstone in precision nutrition by the means of which blood glucose levels are modulated and, in this manner, reducing the risk of developing diabetes mellitus [145]. Precision nutrition offers a novel therapeutic strategy, seeking to identify essential microbiome characteristics, by understanding the complex interactions between the host, the microbiome and dietary factors, in order to predict individual responses to specific food components and, as a result, helping in the process of reshaping the gut microbiota from a disease-associated state to a more balanced and stable condition, thus, unlocking a new chapter in medicine [148].

One study, that assessed recent advances in personalized medicine, describes how maternal nutrition during pregnancy plays a crucial role in the healthy development of the offspring, how gene–nutrient interactions, especially vitamin B12, modulate metabolic risk and the importance of microbial enterotypes in personalized obesity management [149]. Our understanding of the mechanisms behind the individual nutritional requirements variations remains limited; however, the sequencing of the human genome has highlighted the pivotal role of genes in interindividual differences. Furthermore, evidence of diet–gene interactions that influence phenotype highlighted the importance of personalized nutrition mediated by the gut microbiome [150]. Implementing personalized nutrition in the prevention of GDM begins with identifying women at a higher risk of GDM, including those with obesity, advanced maternal age or a family history of diabetes. Furthermore, advances in genetic and metabolic profiling allow for the identification of genetic predispositions that may influence how people digest macronutrients and identify the microbial species, their relative abundance, diversity and functional potential [151]. These provide insights into each individual unique organism and should be complemented with clinical evaluation and other diagnostic tools. Personal recommendations for macronutrient distribution, aligned with ethnic and cultural considerations should be done to prevent postprandial glucose spikes [152]. Tailoring recommendations based on physical activity, stress management and sleep patterns is a key factor for glycemic control, particularly in the prevention and control of conditions like gestational diabetes mellitus [153]. Nutrition counselling and individualized education addresses barriers of healthy eating and empowers women to make informed food choices.

### 6.2. Emerging Microbiota-Based Therapies

This evolving research field holds an unlimited potential for providing a scientific foundation of a more sophisticated approach to personalized nutrition, enabling its effective use by healthcare providers and transforming these insights into highly specific diagnostic tools and precisely targeted therapeutic interventions.

Probiotics (e.g., *Bifidobacterium* and *Lactobacillus*) and prebiotics (e.g., fructooligosaccharides, inulin and galactooligosaccharides) are widely researched functional components that impact gut microbiome interactions, playing an important role in maintaining the balance of its composition [154]. A prebiotic-driven modulation of the gut microbiome does not completely resolve intestinal permeability, endotoxemia or inflammation but it significantly contributes to reductions in body weight and fat deposition, enhancing glucose homeostasis, including glucose tolerance and insulin sensitivity, and optimizing lipid metabolism and leptin responsiveness [155]. Combinations of probiotics and prebiotics are known as synbiotics. Their synergic effect helps in modulating the immune response, in improving gut barrier function and in enhancing digestive health and microbiota balance, showing potential benefits in various conditions such as obesity, IBD and diabetes mellitus [156,157].

Microbial enterotype assessments may improve precision nutrition by integrating the nutritional preferences of individuals with the specific needs of the microbial community of the host, facilitating sustainable weight maintenance and greater adherence to dietary regimens [149]. As a further matter, genetically engineered bacteria represent a promising approach to modulate the gut microbiota, being designed to express therapeutic agents that enhance satiety and boost sensitivity to leptin, while also serving as a delivery system for therapeutic molecules targeting obesity-related conditions that are otherwise challenging to administer [158].

Fecal microbiota transplantation also offers an encouraging strategy in restoring balance in the human gastrointestinal microbiota. This medical procedure involves transferring healthy donor microbiota into a disrupted ecosystem and has great potential as an auxiliary therapy for obesity and in treating recurrent *Clostridioides difficile* infection as well as many other conditions that are yet to be studied [159,160].

### 6.3. Gaps in Research

Research gaps are crucial to the advancement of knowledge in linking diet–gut microbiota to GDM; they need to be identified and addressed with the aim of clarifying and developing further solutions. First and foremost, despite the commitment of researchers, most existing studies involve small cohorts or are observational; thus, the scarcity of large-scale randomized controlled trials that examine the impact of specific dietary patterns on gut microbiota and gestational diabetes mellitus is limiting the applicability of the findings [161,162].

Secondly, most research to date has focused on short-term outcomes without evaluating how these interventions impact maternal metabolic health and the long-term health of the child; as a result, there is a lack of comprehensive understanding as to how these interventions shape short- and long-term risks related to pregnancy, such as the development of diabetes mellitus or obesity, both in mother and their offspring [163,164].

Thirdly, underscoring the individual variability of gut microbiota and its response to dietary interventions, modulated by factors such as genetics, pre-existing metabolic conditions and environmental influence, it needs to be stated that these are not yet well studied in the context of GDM, suggesting the need for personalized nutritional approaches tailored to an individual’s unique microbiota composition [165,166]. While promising and increasingly popular, personalized nutrition faces several limitations that need to be addressed. The complex variability of individual response, high cost of advanced diagnostic tools and continuous need for monitoring and adjustments may not always be feasible for the average person [167]. Moreover, while there is growing research, there is still a limited availability of robust evidence-based information to guide nutrition interventions in the prevention and treatment of gestational diabetes mellitus.

As a final point, more research is needed to unravel the precise mechanisms by which the metabolites influence glucose metabolism and insulin sensitivity during pregnancy, in order to offer more detailed knowledge on how gut microbiome changes contribute to GDM pathophysiology. This information must be integrated and used for the prevention and treatment of pregnant women at risk of or diagnosed with gestational diabetes mellitus. In this matter, information leaflets with simplified information, a balanced view on nutrition and evidence-based recommendations are an essential tool for public education to leverage effective knowledge and improve public health outcomes.

## Figures and Tables

**Table 1:** Selected nutritional intervention studies in gestational diabetes mellitus.

Assessed Dietary Intervention	Ref.	Type of Study	Methods	No. of Participants	Results
Probiotic supplementation	Luoto et al. (2010) [136]	Double-blind placebo-controlled randomized trial	Probiotic supplement containing *Lactobacillus rhamnosus GG* and *Bifidobacterium lactis Bb12*	256	This study demonstrated lower incidence of GDM compared to the control group without perinatal deaths or serious adverse incidences in mothers/newborns.
	Asemi et al. (2013) [137]	Randomized controlled clinical trial	Probiotic yoghurt prepared with cultures of Streptococcus *thermophilus* and *Lactobacillus bulgaricus* and enriched with two strains of lactobacilli (*Lactobacillus acidophilus LA5*) and bifidobacteria (*Bifidobacterium animalis BB12*)	70	The outcome of the study was that daily consumption of probiotic yogurt might help pregnant women prevent developing insulin resistance by maintaining insulin levels.
	Lindsay et al. (2015) [138]	Double-blind placebo-controlled randomized trial	Probiotic supplement containing *Lactobacillus salivarius*	149	Supplementation with probiotic capsules among women with abnormal glucose tolerance had no impact on glycemic control.
	Jafarneiad et al. (2016) [139]	Randomized clinical trial	Probiotic supplement containing 112.5 × 109 CFU/capsule of eight strains of lactic acid bacteria (*Streptococcus thermophilus*, *Bifidobacterium breve*, *Bifidobacterium longum*, *Bifidobacterium infantis*, *Lactobacillus acidophilus*, *Lactobacillus plantarum*, *Lactobacillus paracasei* and *Lactobacillus delbrueckii* subsp. *Bulgaricus*)	89	Probiotic supplementation may have a slightly favorable effect on glycemic status as the product did not significantly affect FPG and HbA1c but prevented the rise in serum insulin concentration and increase in insulin resistance. Therefore, it improved intestinal permeability function and regulated concentration of proinflammatory mediators.
	Kijmanawat et al. (2019) [140]	Double-blind randomized controlled trial.	Probiotic supplements containing *Bifidobacterium* and *Lactobacillus*	57	Probiotic supplements in women with diet-controlled gestational diabetes in the late second and early third trimester had positive effects on fasting glucose levels and increased insulin sensitivity; therefore, they may be considered as an adjunct treatment for glycemic control in these patients.
Synbiotic supplementation	Taghizadeh et al. (2014) [141]	Randomized placebo-controlled	Synbiotic food consisting of a probiotic *Lactobacillus sporogenes*, inulin isomalt, sorbitol and stevia	52	This study illustrated that consumption of synbiotic food in pregnant women improved the insulin response compared to the control food; however, it had no effect on fasting plasma glucose and serum hs-CRP concentrations.
	Ahmadi et al. (2016) [142]	Randomized, double-blind, placebo-controlled trial.	Probiotic supplementation with *Lactobacillus acidophilus*, *Lactobacillus casei* and *Bifidobacterium bifidum* (2 × 109 colony-forming units/g each) plus 800 mg inulin	70	Synbiotic supplementation in GDM women was associated with a significant reduction in serum TAG and VLDL-cholesterol concentrations, but did not influence lipid profiles or PFG.
Studies on gut microbiota in pregnancy	Koren et al. (2012) [30]	Cohort study	Stool samples (from T1 and T3 of pregnancy as well as woman’s infants at 1 month of age, 6 months of age and 4 years of age), diet information and clinical data	91	During pregnancy, gut microbiota reshapes, particularly in the third trimester, resembling a disease-associated state (dysbiosis) that differs among women, having an increased number of Proteobacteria and Actinobacteria species. These microbial shifts were linked to increased insulin resistance and higher inflammatory response.
	Miller et al. (2021) [143]	Longitudinal cohort study	Adherence to Mediterranean diet pattern was scored by the Alternate Mediterranean Diet Quality Score	41	Mediterranean diet pattern is associated with greater diversity of the microbiota community, promoting the production of SCFAs.
	Su et al. (2021) [144]	Cohort study	Fecal microbiota profiles from women with GDM normoglycemic women were assessed by 16S rRNA gene sequencing; fasting metabolic hormone concentrations were measured using multiplex ELISA.	53	Dysbiosis of the gut microbiome exists in patients with GDM in the second trimester of pregnancy; specifically, the phylum Bacteroidetes increased in GDM, as did *Bacteroides*, *Incertae sedis*, *Citrobacter*, *Parabacteroides*, and *Fusicatenibacter* genus. There are connections between gut microbiome and glucose plasma levels; thus, it might be possible that dysbiosis can be involved in the pathogenesis of GDM revealing the potential of these biomarkers in prevention and intervention strategies in GDM.
